# Accuracy of Proton Magnetic Resonance Spectroscopy in Distinguishing Neoplastic From Non-neoplastic Brain Lesions

**DOI:** 10.7759/cureus.49824

**Published:** 2023-12-02

**Authors:** Vudem Ranjith Kumar Reddy, Satyanarayana Kummari, Kiran Goud Burra, Saraswata Das

**Affiliations:** 1 Department of Radiology, Government Medical College, Siddipet, Siddipet, IND; 2 Department of Radiology, Great Eastern Medical School & Hospital, Ragolu, IND; 3 Department of Radiology, Government District Hospital Medak, Medak, IND; 4 Department of Radiodiagnosis, College of Medicine and JNM Hospital, Kalyani, IND

**Keywords:** lipid and lactate peak, mrs, magnetic resonance spectroscopy, neoplastic lesion, non-neoplastic lesions, naa, choline, mri- magnetic resonance imaging, neoplastic, brain lesions

## Abstract

Objective: To evaluate the advantage of a combination of magnetic resonance spectroscopy (MRS) and magnetic resonance imaging (MRI) over MRI in the diagnosis of intracranial mass lesions to differentiate between neoplastic and non-neoplastic lesions and compare them with histopathology and clinical data as gold standard.

Methodology: This was a descriptive cross-sectional study conducted at the Department of Radiology, Apollo Hospital located in Jubilee Hills, Hyderabad. In the present study, a total of 60 patients of all ages with brain masses found through MRI with positive clinical symptoms, regardless of gender, were included. We also involved patients with non-brain cancers suspected of spreading to the brain.

Result: MRI identified 63% of lesions as neoplastic and 37% as non-neoplastic. Combining MRI and MRS increased accuracy, with 65% of the lesions diagnosed as neoplastic and 35% as non-neoplastic, demonstrating that MRS significantly enhances diagnostic precision compared to MRI alone.

Conclusion: This study aimed to see how combining MRI and MRS helps diagnose brain masses, comparing with histopathology as the gold standard. MRI alone identified 63% as neoplastic, but MRI with MRS improved accuracy (65%). MRI sensitivity was 87.80%, but combined with MRS, it increased to 92.68%. Thus, the study concluded that the combination of MRI and MRS is more accurate than MRI alone.

## Introduction

Among the various emerging magnetic resonance (MR) techniques being employed in clinical settings, proton MR spectroscopy (1H-MRS) stands out due to its distinctive ability to offer a chemical-pathological assessment of lesions that are visible in MR images, as well as normal-appearing brain tissues [1]. During the early 1980s, MR emerged as a valuable tool for examining human anatomy, physiology, and biochemistry [2]. Unlike MR imaging (MRI) and MR angiography (MRA), which primarily focus on the physical aspects of the human body, in vivo MR spectroscopy (MRS) delves into metabolic pathways and their stable conditions in various tissue types [3]. 1H-MRS enhances the value of MRI by supplying clinically pertinent details about metabolites in typical brain irregularities [4]. It is now in clinical settings for diagnosing, predicting outcomes, and evaluating treatments for a range of conditions, such as brain tumors, neonatal and pediatric disorders, demyelinating disorders, and infectious brain lesions [5]. It is expected to play a significant role in managing neurodegenerative disorders, epilepsy, and stroke [6]. Clinical MRS can be effectively performed at either 1.5 or 3.0 tesla when using reproducible protocols adhering to quality standards. However, to gain broader clinical acceptance, there is a need to harmonize data acquisition and processing procedures across different vendors due to the current lack of standardization and quality assurance in MRS methods [7]. The overall incidence ranges from five to 13 cases per 100,000 people [8]. MRS is a non-invasive and very sensitive method, but it lacks the necessary specificity to consistently differentiate between brain lesions that are cancerous and those that are not. Therefore, it should be regarded as a complementary tool to traditional imaging methods like T1, T2, fluid-attenuated inversion recovery (FLAIR), and contrast sequences rather than a substitute for histopathological examination [9].

MRS differentiates neoplastic from non-neoplastic lesions by assessing specific criteria, including ratios like choline (Cho)/creatine (Cr) and Cho/N-acetylaspartate (NAA), as well as the presence of choline and NAA peaks on the MR spectrum [10]. In instances involving brain lesions, deviations in metabolite levels are detected when compared to normal tissue [11]. For instance, infectious lesions such as brain abscesses typically exhibit decreased levels of major metabolites, including Cho, NAA, and Cr, whereas neoplastic lesions often display an elevated Cho peak along with reduced NAA levels [12]. The literature highlights the growing utility of MRS in the examination of brain tumors, infections, and various types of inflammatory lesions. Recent reports reinforce the effectiveness of MRS as a potent tool for grading gliomas and distinguishing them from metastatic lesions [13].

The main aim of the study was to assess the clinical utility of 1H-MRS added to MRI for the differentiation of intracranial neoplastic using histopathology as the gold standard and non-neoplastic mass lesions like abscess, toxoplasma, tuberculomas, and infarcts diagnosed by microbiological and clinical data.

## Materials and methods

In a descriptive cross-sectional study, we assessed the efficacy of MRS as a diagnostic tool for brain lesions. This investigation took place at the Department of Radiology, Apollo Hospital, located in Jubilee Hills, Hyderabad from May 2017 to June 2018 (13 months). The study was approved by the Hospital Ethics Committee. The combination of MRI and MRS results provides good results diagnosis of intracranial mass lesions (to differentiate neoplastic from non-neoplastic mass lesions). In this study, a total of 60 participants were included.

Patients of all ages and genders who had brain masses based on routine MRI and clinical features and patients who had primary non-CNS neoplasms with suspicion of secondaries in the brain were included in this study. Patients with brain aneurysm clips, implanted neural stimulators, cardiac pacemakers, cochlear implants, ocular foreign bodies, metal shrapnel, or other medical devices inside their bodies, as well as those who have undergone surgeries where metal clips or wires cannot be ruled out, were excluded from this study.

In this research, a choline/NAA ratio of 2.2 was employed to differentiate primary high-grade neoplasms from conditions that resemble them, despite some overlapping MR spectroscopic ratios seen in cases of encephalitis, tumefactive demyelinating lesions, and gliomas.

Patient screening and inclusion in the study

Patients who met the study's inclusion criteria were informed about the study, and their informed consent was obtained. A study proforma was completed for each eligible patient. After patient positioning, a global shimming procedure was conducted to optimize the magnetic field homogeneity across the entire volume detected by the receiver coil. Global shimming provided an initial reference point for local shimming. Subsequently, MR images were acquired for localization purposes. Images were obtained in all three planes (coronal, axial, and sagittal) to guide the placement of a voxel. In cases where the patient remained still, MR images acquired during routine imaging were utilized for localization. Next, specific MRS parameters were selected, with a focus on important factors such as TR (repetition time) and TE (echo time). A volume of interest (VOI) was carefully defined, and local shimming was performed to optimize homogeneity within the selected volume. To enhance the visibility of smaller metabolite peaks, the water peak was suppressed using the chess (chemical shift selective spectroscopy) technique. Subsequently, MRS data were collected. The acquired data were then processed to generate spectra and spectral maps. The zero point of the spectrum was calibrated within the software using the frequency of a particular compound known as tetramethylsilane (TMS). Following data interpretation, each patient's findings were correlated with histopathologic results, and data entry was conducted into a Microsoft Excel spreadsheet (Microsoft Corporation, Redmond, WA) for further analysis.

Statistical analysis

Data analysis was conducted using Microsoft Excel and SSPS software version 21 (IBM Corp., Armonk, NY). The analysis included preliminary data inspection, data mining, and interpretation. Categorical variables like age, sex, and family history were represented using frequencies and percentages, while MRS findings such as the choline/NAA ratio were expressed as means and standard deviations. We calculated sensitivity, specificity, positive predictive value, and negative predictive value to assess the diagnostic accuracy of both MRI and MRI + MRS. Statistical significance was determined with a threshold of p-values less than 0.05.

## Results

In this study, 55% of the participants were male, while 45% were female. The mean age was 34.40 years, and the standard deviation was 24.10 years. A significant portion of the participants (40%) belonged to the age group above 40 years, and 26.7% were between one and 10 years (Table 1).

**Table 1:** Distribution of gender and age group

Gender	Frequency	Percentage
Females	27	45.00%
Males	33	55.00%
Total	60	100.00%
Age group in years		
1-10	16	26.70%
11-20	5	8.30%
21-30	6	10.00%
31-40	9	15.00%
>40	24	40.00%
Total	60	100.00%

According to the MRI findings, 63% of the lesions were identified as neoplastic, while 37% were categorized as non-neoplastic. When utilizing both MRI and MRS techniques, 65% of the lesions were diagnosed as neoplastic, with the remaining 35% classified as non-neoplastic (Table 2).

**Table 2:** Diagnosis of lesions on MRI and MRI with MRS MRS: magnetic resonance spectroscopy.

MRI	Frequency (n)	Percentage	P-value
Neoplastic	38	63.30%	P = 0.001
Non-neoplastic	22	36.70%	
Total	60	100.00%	
MRI + MRS			
Neoplastic	39	65.00%	P = 0.001
Non-neoplastic	21	35.00%	
Total	60	100.00%	

**Figure 1:**
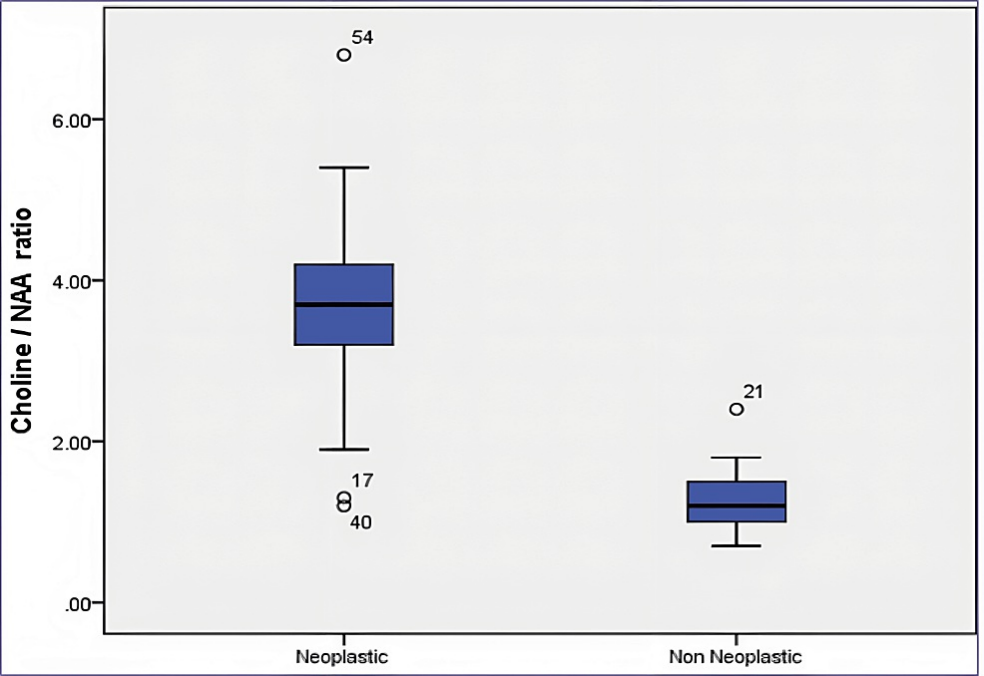
Choline/NAA ratio NAA: N-acetylaspartate.

In neoplastic cases, the mean choline/NAA ratio is 3.62 with a standard deviation of ±1.40, indicating an average higher concentration of choline relative to NAA in these tumor-related tissues. Conversely, in non-neoplastic cases, the mean choline/NAA ratio is 1.29 with a standard deviation of ±1.40, demonstrating a lower average ratio in tissues without tumors (Figure 1).

The diagnostic tests, involving MRI alongside biopsy and clinical data to differentiate neoplastic and non-neoplastic cases, yielded promising results. When relying on MRI with biopsy and clinical information, 53 out of 60 cases were accurately identified (36 neoplastic, 17 non-neoplastic). Introducing MRS improved accuracy further; out of the same 60 cases, 57 were correctly identified (39 neoplastic, 18 non-neoplastic) when using both MRI and MRS. This indicates that adding MRS to the diagnostic process significantly enhanced accuracy, making it a more reliable method compared to MRI alone (Table 3).

**Table 3:** Biopsy and clinical data with only MRI and MRI with MRS MRS: magnetic resonance spectroscopy.

MRI	Biopsy and clinical data			Total
	Neoplastic		Non-neoplastic	
Neoplastic	36		2	38
Non-neoplastic	5		17	22
Total	41		19	60
Biopsy and clinical data				
MRI + MRS	Neoplastic	Non-neoplastic		
Neoplastic	38		1	39
Non-neoplastic	3		18	21
Total	41		19	60

The accuracy of MRI in comparison to the gold standard is noteworthy, with a sensitivity of 87.80% and a specificity of approximately 89.47%. Additionally, the positive predictive value stands at 94%, and the negative predictive value hovers around 88%. When both MRI and MRS are taken into account in relation to the gold standard, the sensitivity increases to 92.68%, and the specificity is around 94.74%. Moreover, the positive predictive value is 85%, while the negative predictive value is approximately 93%. Combining MRI with MRS significantly enhances diagnostic capabilities compared to using MRI alone, showcasing superior sensitivity and specificity. This indicates that the joint utilization of MRI and MRS forms a robust diagnostic tool, demonstrating impressive accuracy in identifying and distinguishing various medical conditions (Table 4).

**Table 4:** Sensitivity, specificity, positive predictive value, and negative predictive value of MRI MRS: magnetic resonance spectroscopy; PPV: positive predictive value; NPV: negative predictive value.

Statistic	MRI		MRI + MRS	
	Value	95% CI	Value	95% CI
Sensitivity	87.80%	73.80% to 95.92%	92.68%	80.08% to 98.46%
Specificity	89.47%	66.86% to 98.70%	94.74%	73.97% to 99.87%
PPV	94.74%	82.84% to98.53%	97.44%	84.91% to 99.61%
NPV	77.27%	59.58% to 88.69%	85.71%	66.76% to 94.72%
Accuracy	88.33%	77.43% to 95.18%	93.33%	83.80% to 98.15%

## Discussion

**Figure 2:**
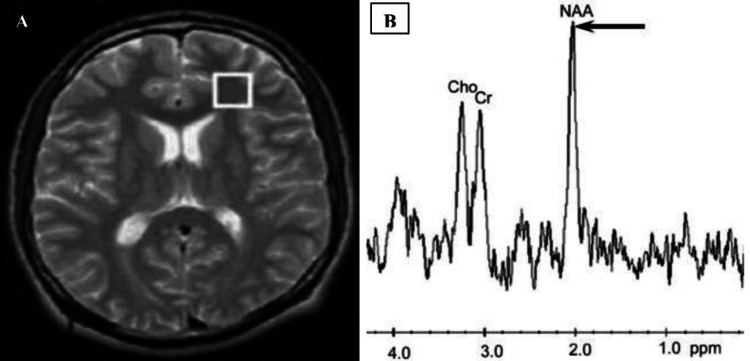
Proton MRS of cerebral parenchyma in a normal volunteer A: Axial T2-weighted image with voi is placed in the left frontal lobe. B: The spectrum demonstrates prominent NAA, Cr, and Cho. MRS: magnetic resonance spectroscopy; NAA: N-acetylaspartate; Cho: choline; Cr: creatine.

In our study with MRI alone, 63% of the lesions were identified as neoplastic, while 37% were categorized as non-neoplastic. When utilizing both MRI and MRS techniques, 65% of the lesions were diagnosed as neoplastic with the remaining 35% classified as non-neoplastic (Table 2). In the present study, the histopathological evaluation revealed that 41 (68%) cases were neoplastic, while 19 (32%) were non-neoplastic lesions (Table 3). In the current study, the mean choline/NAA ratio in neoplastic lesions was significantly high compared to the mean ratio in non-neoplastic lesions. Our results contrast with the study done by Jesrani et al. [14]. They revealed histopathological analysis and reported that 43.1% of cases were neoplastic, while 56.9% were non-neoplastic. Similarly, the study identified neoplastic lesions as those characterized by reduced NAA levels and frequently elevated Cho levels, leading to increased Cho/NAA ratios (Figure 1). Gender distribution is given in Table 1. Proton MRS of cerebral parenchyma in a normal volun­teer demonstrated prominent NAA, Cr, and Cho (Figure 2).

**Figure 3:**
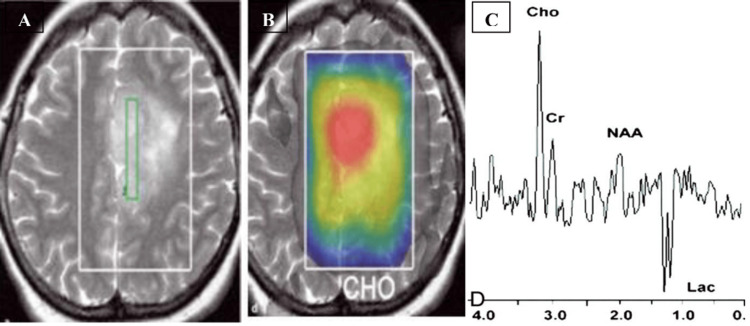
Proton MRS in a patient with a glioma. A: Axial T2-weighted image shows increased signal intensity in the mass with minimal peritumoral edema. B: Color map of choline metabolite. C: Spectrum demonstrates markedly elevated Cho and decreased NAA with a Cho/NAA ratio of 2.60, as well as increased lactate (Lac). MRS: magnetic resonance spectroscopy; NAA: N-acetylaspartate; Cho: choline; Cr: creatine.

Alshammari et al. [15] effectively showed elevated Cho/NAA and Cho/Cr ratios in gliomas, astrocytomas, and other malignant tumors. Notably, meningioma and malignancy exhibited a significant increase in both lipid and lactate peaks in addition to Cho. High-grade gliomas were characterized by increased Cho levels, decreased NAA and Cr levels, and elevated lactate levels (Figure 3).

**Figure 4:**
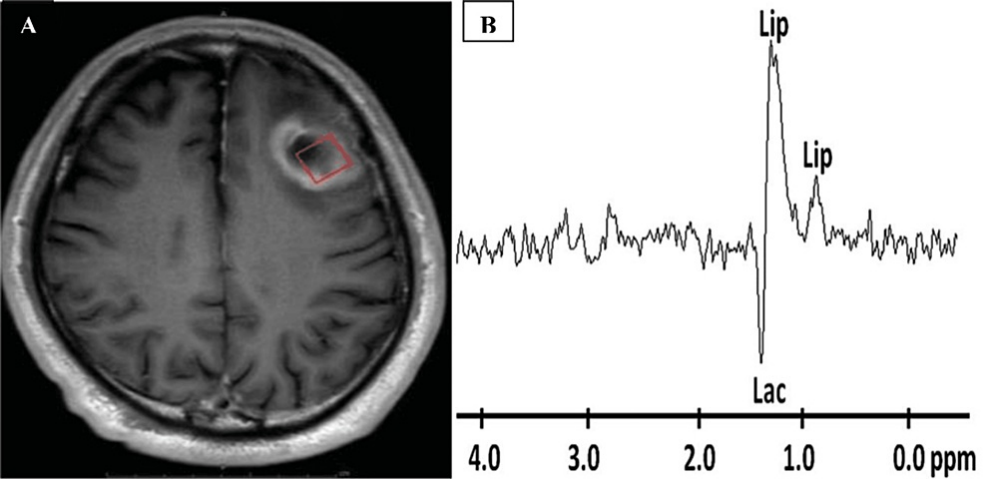
Proton MRS in a patient with brain abscess A: Axial Gd-enhanced T1-weighted image of brain abscess in the left frontal lobe. B: Spectrum demonstrates Lip/Lac peaks 1.3 ppm. MRS: magnetic resonance spectroscopy.

The increased choline levels in brain tumors were linked to heightened mitotic activity, which was not observed in non-neoplastic lesions. Typically, neoplastic brain lesions displayed elevated choline levels, reduced NAA levels, and the presence of lipid or lactate peaks, which are not commonly found in normal brain conditions. MRS in a patient with brain abscess demonstrated lipid/lactate peaks (Figure 4).

In the present study, the sensitivity of MRI and MRS was noted at 92.68% and specificity was found to be 94.74% against the sensitivity of 87.8% and specificity of 89.47% with MRI alone (Table 4). MRI along with MRS is a good diagnostic tool when compared to MRI alone, as it has good sensitivity and specificity. This was comparable with the study conducted by Jesrani et al. [14]. The findings of their study show a sensitivity of 87.5%, specificity of 93.3%, and accuracy of 92.1%. Whereas, Alshammari et al. [15] demonstrated MRS sensitivity of 82.60%, specificity of 85.71%, and accuracy of 100% in effectively distinguishing between neoplastic and non-neoplastic brain lesions.

Similarly, another study was performed on 176 patients who were presented with focal intracranial mass lesions to the neuroradiology department. After initial structural MRI and/or CT scans, all patients underwent a consistent single-volume 1H-MRS protocol. The study examined 176 lesions, most of which were histologically confirmed, using a standardized 1H-MRS protocol available for clinical use. The author revealed that incorporating spectroscopy alongside MRI aids in characterizing tissue in intracranial mass lesions, ultimately enhancing the accuracy of diagnosing focal brain diseases [16].

In another study by Majos et al. [17], they involved a diverse patient population, which included individuals with both previously known tumors and those with newly discovered masses of uncertain origin, the diagnostic performance of MRS exhibited variability. Sensitivities ranged from 79% to 100%, and specificities varied between 74% and 100%. The positive predictive values spanned from 92% to 100%, while the negative predictive values ranged from 60% to 100%.

Also, Butzen et al. [18], in their research involving 99 cases, consisting of 86 neoplasms and 13 non-neoplastic cases, achieved a specificity of 87%. They also obtained a similar sensitivity of 85% when employing the Cho/NAA ratio threshold of 1 for distinguishing neoplastic from non-neoplastic brain lesions using MRI-guided single-voxel proton MRS data.

MRS is a notably sensitive and specific approach for differentiating between neoplastic and non-neoplastic brain lesions. It serves as a valuable adjunct in challenging cases, with Cho, Cho/Cr, and Cho/NAA ratios emerging as the most reliable indicators in MRS for differentiation. Imaging helps make two important differentiations: one between high-grade and low-grade tumors and the other between neoplastic and non-neoplastic lesions. Identifying the tumor grade preoperatively is particularly significant, as high-grade tumors often require more aggressive treatment approaches.

Our study has a few limitations. A small number of patients were examined. It is a short-duration study. We did not include the patients who were lost to follow-up. However, a longer study including a larger number of patients would give a true picture of the problem.

## Conclusions

MRS has demonstrated its reliability and accuracy in distinguishing between neoplastic and non-neoplastic brain lesions. Combining MRI and MRS is beneficial for diagnosing intracranial masses, using histopathology, microbiological, and clinical data as standards. MRI alone identified 63% as neoplastic and 37% as non-neoplastic. MRI + MRS increased neoplastic diagnoses to 65% and non-neoplastic to 35%. Biopsy confirmed 68% of tumors as malignant (p = 0.001). MRI had 87.80% sensitivity and 89.47% specificity. MRI + MRS had 92.68% sensitivity and 94.74% specificity. Combining MRI and MRS proved valuable, offering higher accuracy than MRI alone.
